# Peritoneal Inclusion Cyst Mimicking Peritoneal Carcinomatosis: A Benign Lesion Posing a Malignant Dilemma

**DOI:** 10.7759/cureus.97290

**Published:** 2025-11-19

**Authors:** Veena Mudaliar, Pubudu Piyatissa, Rupesh Rajendran, Tauseef Ashraf, Rida Fatima

**Affiliations:** 1 Department of Undergraduate Medical Education, Pilgrim Hospital, United Lincolnshire Hospitals NHS Trust, Boston, GBR; 2 Department of Radiology, Pilgrim Hospital, United Lincolnshire Hospitals NHS Trust, Boston, GBR; 3 Department of Internal Medicine, Freeman Hospital, Newcastle upon Tyne Hospitals NHS Foundation Trust, Newcastle upon Tyne, GBR; 4 Department of General Surgery, Pilgrim Hospital, United Lincolnshire Hospitals NHS Trust, Boston, GBR

**Keywords:** benign mesothelial cyst, ct imaging, diagnostic mimicry, omental nodularity, pelvic cystic lesions, peritoneal carcinomatosis, peritoneal inclusion cyst, radiologic pathologic correlation, reactive mesothelial proliferation

## Abstract

Peritoneal carcinomatosis is typically associated with advanced malignancy and is most often identified on imaging by multifocal peritoneal and omental involvement. However, benign conditions may closely mimic these findings, leading to diagnostic uncertainty. Peritoneal inclusion cysts are benign, multiloculated cystic lesions arising from reactive mesothelial proliferation, often in the context of prior inflammation or adhesions, and may resemble peritoneal carcinomatosis on cross-sectional imaging.

We describe a case of a 68-year-old postmenopausal woman with a history of diverticulitis who presented with chronic non-offensive vaginal discharge and intermittent hematuria. Pelvic examination revealed no abnormal findings, and serum CA-125 was within the normal range. Pelvic magnetic resonance imaging showed a loculated fluid collection in the pouch of Douglas, and contrast-enhanced computed tomography identified multiple peritoneal and omental nodules, raising concern for peritoneal carcinomatosis. Diagnostic laparoscopy and subsequent total abdominal hysterectomy with bilateral salpingo-oophorectomy and infracolic omentectomy were performed. Histopathological evaluation confirmed benign multicystic peritoneal inclusion cysts without atypia. This case underscores the diagnostic challenge when benign peritoneal pathology mimics malignancy. When tumor markers are normal and no primary malignancy is identified, careful integration of clinical assessment, imaging features, and histopathologic confirmation is essential to avoid unnecessary radical intervention.

## Introduction

Peritoneal carcinomatosis most commonly arises from advanced gastrointestinal or gynecologic malignancies. It is characterized by the dissemination of malignant cells throughout the peritoneal cavity, often presenting radiologically with peritoneal thickening, omental caking, and ascites [1-3]. However, several benign conditions can closely mimic these features, leading to diagnostic uncertainty. These include peritoneal inclusion cysts (PICs), endometriosis, tuberculosis-related peritonitis, and other reactive or inflammatory processes [4-6].

PICs are benign, multiloculated cystic lesions resulting from reactive mesothelial proliferation, typically occurring in the setting of prior pelvic surgery, trauma, or inflammatory processes that lead to peritoneal adhesions [7]. Although they are most frequently encountered in premenopausal women, PICs can also occur in postmenopausal patients, where their appearance may raise a stronger suspicion for malignancy. Recognizing this possibility is clinically important because benign peritoneal lesions may radiologically resemble metastatic disease, potentially leading to diagnostic confusion and unnecessary invasive treatment if not carefully evaluated. We present this case to highlight how peritoneal inclusion cysts can mimic peritoneal carcinomatosis on cross-sectional imaging, emphasizing the importance of radiologic-clinical correlation and histopathologic confirmation to avoid overtreatment.

## Case presentation

A 68-year-old postmenopausal woman with a remote history of right-sided diverticulitis was referred to gynecologic services with concern for possible postmenopausal bleeding. On further discussion, she clarified that she had not experienced vaginal bleeding but rather intermittent hematuria, which was undergoing urologic investigation. She also reported a chronic non-offensive whitish vaginal discharge requiring panty liners, without systemic symptoms, abdominal pain, weight loss, or gastrointestinal disturbance. Pelvic examination demonstrated a normal vulva, vagina, and cervix with mild atrophic features, and a high vaginal swab was negative. Serum CA-125 was 17 U/mL (within the normal range).

**Figure 1:**
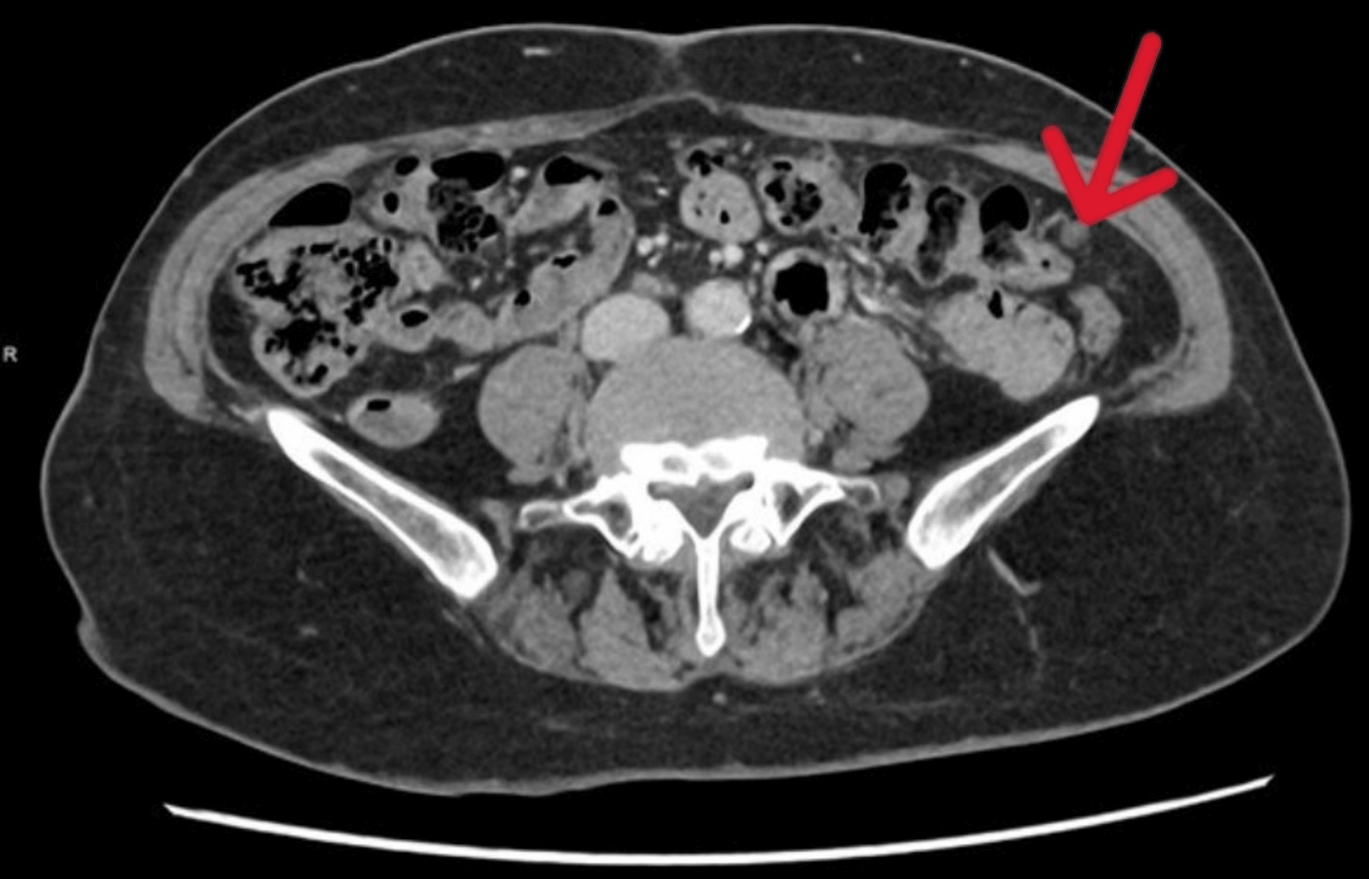
Axial contrast-enhanced CT showing a feeding vessel supplying the peritoneal cyst (arrow).

Transvaginal ultrasound showed an endometrial thickness of 5.9 mm and a right adnexal lesion measuring approximately 6×2 cm. Pelvic MRI demonstrated a loculated fluid collection in the pouch of Douglas, slightly right of midline. To further evaluate this, a contrast-enhanced CT scan of the chest, abdomen, and pelvis was obtained. An axial contrast-enhanced CT image demonstrated cystic peritoneal lesions and suggested a feeding vessel supplying one of the lesions (Figure 1).

The CT further demonstrated multiple small soft-tissue nodules along the peritoneum and omentum, ranging from approximately 0.5-2.5 cm in diameter, with the largest in the right iliac fossa. The appearance was reported as highly suspicious for peritoneal carcinomatosis, despite normal tumor markers.

Diagnostic laparoscopy revealed mucinous peritoneal lesions. However, initial peritoneal and omental biopsies demonstrated no malignancy. Due to persistent diagnostic uncertainty and the extent of disease, a multidisciplinary consensus recommended definitive surgical management. The patient subsequently underwent total abdominal hysterectomy, bilateral salpingo-oophorectomy, infracolic omentectomy, and appendectomy.

**Figure 2:**
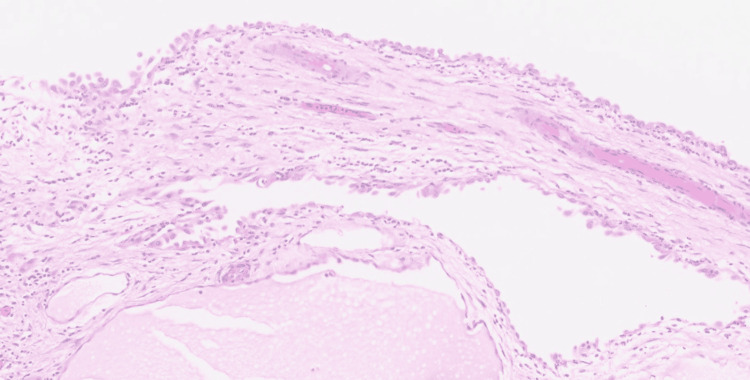
Histopathology (high-power) - benign mesothelial lining confirming inclusion cysts.

**Figure 3:**
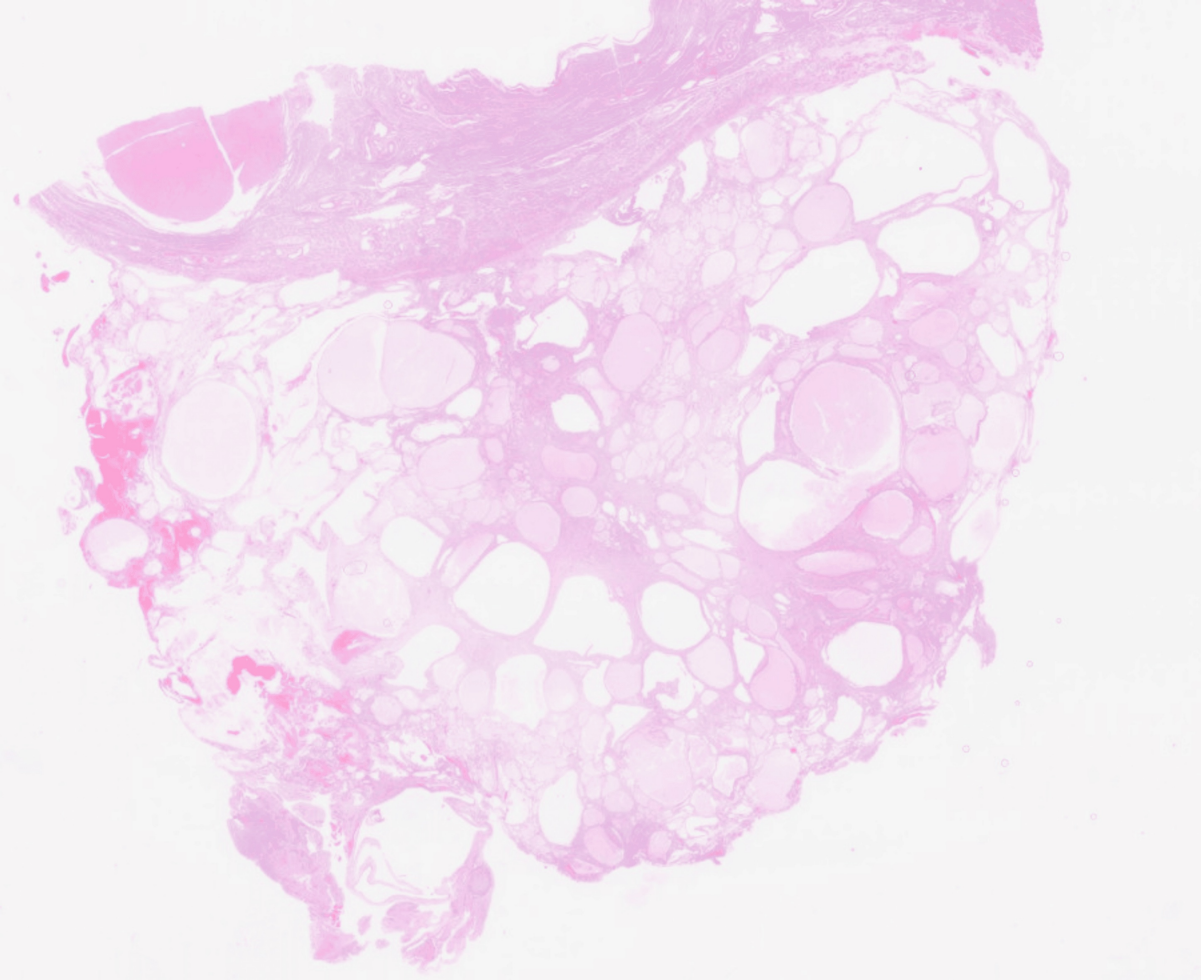
Histopathology (low-power) - inclusion cysts involving posterior uterine serosa.

Histopathologic examination revealed multiple benign multicystic peritoneal inclusion cysts lined by flattened to cuboidal mesothelial cells without atypia or mitosis. Low-power histopathology showed inclusion cysts involving the posterior uterine serosa, and high-power magnification confirmed a benign mesothelial lining consistent with peritoneal inclusion cysts (Figures 2, 3).

**Figure 4:**
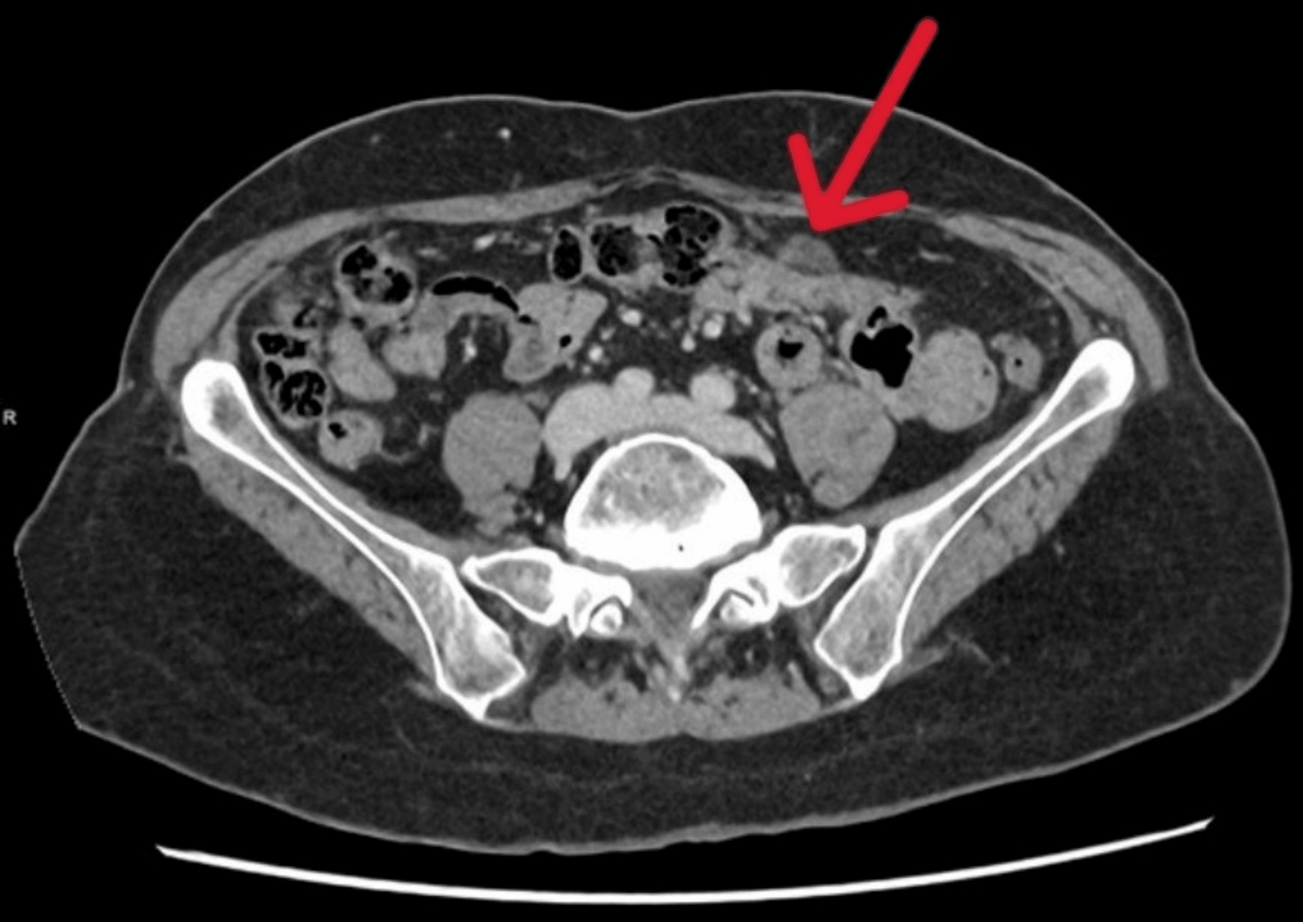
Axial CT image demonstrating cystic peritoneal lesions resembling peritoneal carcinomatosis (arrow).

A separate axial CT image further illustrates how the cystic peritoneal lesions mimicked peritoneal carcinomatosis radiologically (Figure 4). The patient recovered well postoperatively and was discharged with planned follow-up, given the variable risk of recurrence.

## Discussion

Peritoneal inclusion cysts (PICs) are benign, multiloculated cystic lesions formed when reactive mesothelial cells become entrapped by peritoneal adhesions, often following inflammation, infection, endometriosis, or prior pelvic surgery [4,7]. Their appearance can be highly variable, and in some cases, they may closely resemble peritoneal carcinomatosis (PC) on cross-sectional imaging. This overlap can lead to diagnostic uncertainty when imaging suggests diffuse peritoneal involvement, but the clinical context lacks clear evidence of malignancy.

In this case, the patient’s normal serum CA-125 level, absence of systemic symptoms, and lack of an identifiable primary tumor were not initially sufficient to exclude malignancy, as the CT demonstrated multifocal peritoneal and omental nodularity, a pattern classically associated with PC. However, PICs typically demonstrate thin internal septations and a predominantly cystic morphology, without solid enhancing nodules, while PC often presents with soft-tissue implants, irregular peritoneal thickening, and omental caking [1,2]. Recognizing these imaging characteristics is particularly important when tumor markers are normal or no primary malignancy is known.

**Figure 5:**
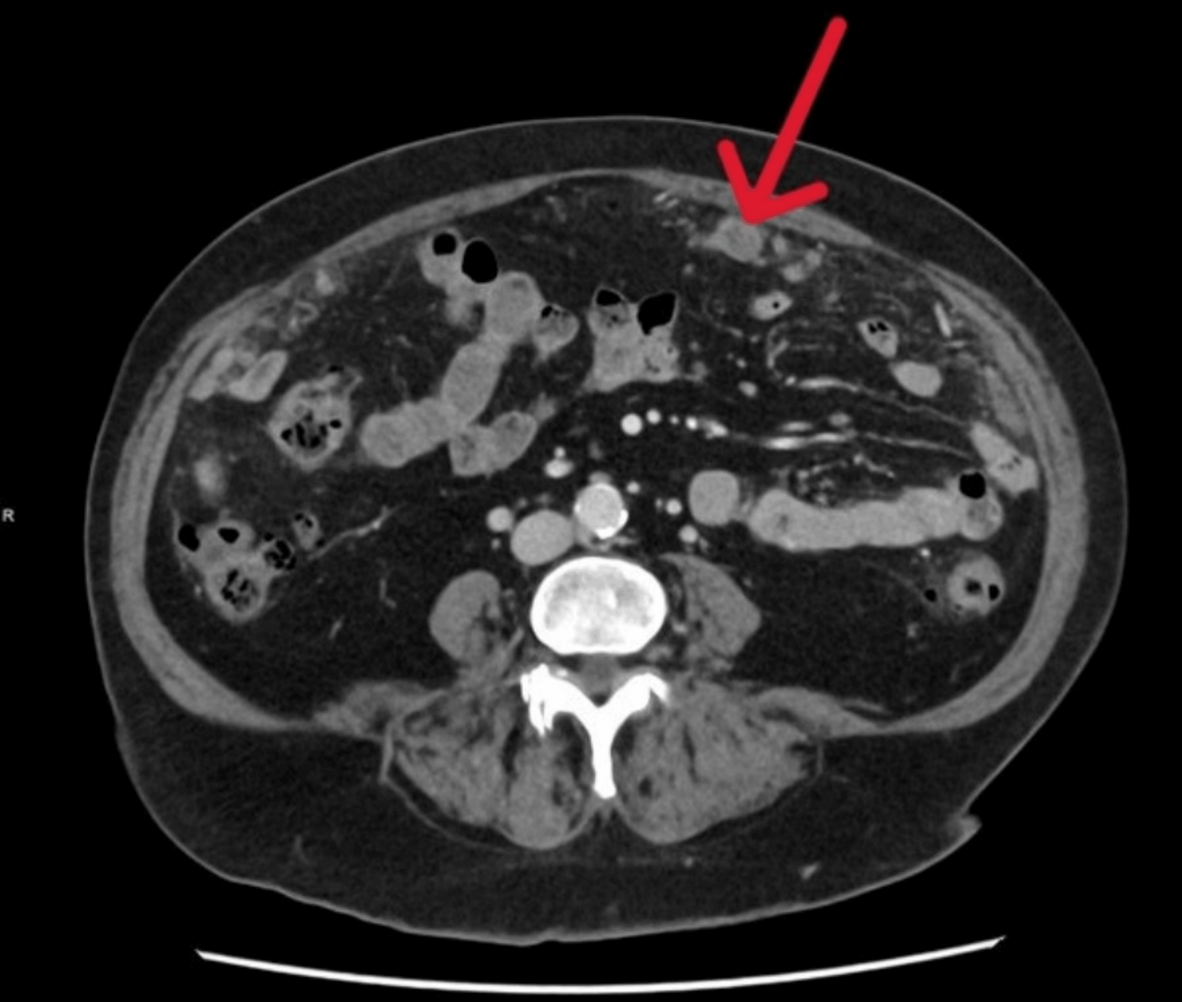
Axial CT image from a different patient demonstrating peritoneal carcinomatosis, included for comparison. The irregular soft-tissue peritoneal implants and omental involvement resemble the cystic lesions in this case (arrow), highlighting the potential for peritoneal inclusion cysts to mimic malignant disease. This image is from a different patient and has been fully de-identified in accordance with institutional and ICMJE privacy standards. ICMJE: International Committee of Medical Journal Editors

Histopathologic analysis remains crucial in distinguishing PICs from malignant peritoneal disease. In this case, pathology demonstrated mesothelial-lined cystic structures without atypia, confirming a benign reactive process [6]. This radiologic-pathologic discrepancy underscores the importance of correlating imaging findings with clinical context and obtaining tissue diagnosis when imaging is equivocal. To illustrate this diagnostic challenge, Figure 5 provides a comparison axial CT image from a different patient with confirmed peritoneal carcinomatosis, demonstrating how PICs can radiologically mimic malignant peritoneal disease.

To assist clinicians in differentiating these conditions, key distinguishing features are summarized in Table 1. These features are adapted from previously described imaging and histopathologic criteria [1,4,5]. Recurrence of PICs has been reported in 30-50% of cases, particularly when excision is incomplete, emphasizing the importance of planned postoperative surveillance and multidisciplinary follow-up [1].

**Table 1:** Comparison of peritoneal inclusion cysts, peritoneal carcinomatosis, and endometriosis. This table highlights differences in clinical presentation, imaging characteristics, histopathology, and management.

Features	Peritoneal inclusion cysts (PICs)	Peritoneal carcinomatosis	Endometriosis
Etiology/pathogenesis	Reactive mesothelial proliferation associated with prior inflammation, surgery, or adhesions	Dissemination of malignant cells from a primary tumor (e.g., ovarian, colorectal, gastric)	Ectopic endometrial tissue implantation and cyclical hormonal response
Typical patient profile	More common in reproductive-aged women, but can occur post menopause	Often known or suspected primary malignancy	Typically, reproductive-aged women, often with dysmenorrhea or infertility
Imaging appearance	Multiloculated cystic lesions with thin septations; no solid enhancing nodules	Soft-tissue nodules, omental caking, ascites; solid or irregular enhancing components	Endometriomas, fibrotic adhesions, and sometimes small peritoneal nodules
Tumor markers	Usually normal (e.g., CA-125 normal or mildly elevated)	Often elevated, depending on primary tumor	Typically, normal, or slightly elevated CA-125
Histopathology	Cysts lined by flattened mesothelial cells without atypia	Malignant epithelial or mesenchymal cells infiltrating peritoneum/omentum	Endometrial glands and stroma outside the uterus
Management	Symptom management or surgical excision if symptomatic or uncertain diagnosis	Systemic therapy and/or cytoreductive surgery	Hormonal therapy or surgical excision, depending on symptoms
Key distinguishing feature	Benign; mimics malignancy radiologically but lacks solid components or atypia	Malignant; presence of solid enhancing masses or diffuse infiltration	Cyclical pain and ovarian endometriomas are often clues

## Conclusions

Peritoneal inclusion cysts can closely resemble peritoneal carcinomatosis on cross-sectional imaging, particularly when multiple peritoneal or omental lesions are present. When cystic morphology, thin septations, and the absence of solid enhancing nodules are identified in a patient without clinical or biochemical evidence of malignancy, benign etiologies, such as PICs, should be considered in the differential diagnosis. Histopathologic confirmation remains essential when imaging findings are equivocal to avoid unnecessary radical intervention. Multidisciplinary collaboration between radiology, surgery, and pathology is central to establishing an accurate diagnosis and guiding appropriate management.
